# Mitochondrial Medicine: Genetic Underpinnings and Disease Modeling Using Induced Pluripotent Stem Cell Technology

**DOI:** 10.3389/fcvm.2020.604581

**Published:** 2021-01-18

**Authors:** Parisa K. Kargaran, Diogo Mosqueira, Tamas Kozicz

**Affiliations:** ^1^Department of Cardiovascular Medicine, Center for Regenerative Medicine, Mayo Clinic, Rochester, MN, United States; ^2^Division of Cancer & Stem Cells, Biodiscovery Institute, University of Nottingham, Nottingham, United Kingdom; ^3^Department of Clinical Genomics, Mayo Clinic, Rochester, MN, United States

**Keywords:** human induced pluripotent stem cells, cardiomyocytes, regenerative medicine, mitochondrial disease, drug discovery, sonar sensor

## Abstract

Mitochondrial medicine is an exciting and rapidly evolving field. While the mitochondrial genome is small and differs from the nuclear genome in that it is circular and free of histones, it has been implicated in neurodegenerative diseases, type 2 diabetes, aging and cardiovascular disorders. Currently, there is a lack of efficient treatments for mitochondrial diseases. This has promoted the need for developing an appropriate platform to investigate and target the mitochondrial genome. However, developing these therapeutics requires a model system that enables rapid and effective studying of potential candidate therapeutics. In the past decade, induced pluripotent stem cells (iPSCs) have become a promising technology for applications in basic science and clinical trials, and have the potential to be transformative for mitochondrial drug development. Engineered iPSC-derived cardiomyocytes (iPSC-CM) offer a unique tool to model mitochondrial disorders. Additionally, these cellular models enable the discovery and testing of novel therapeutics and their impact on pathogenic mtDNA variants and dysfunctional mitochondria. Herein, we review recent advances in iPSC-CM models focused on mitochondrial dysfunction often causing cardiovascular diseases. The importance of mitochondrial disease systems biology coupled with genetically encoded NAD^+^/NADH sensors is addressed toward developing an *in vitro* translational approach to establish effective therapies.

## Introduction

Mitochondria are fundamental structures in eukaryotes since they play a dynamic role in cellular metabolism and are critical for ATP production. However, alterations in mitochondrial function can result in the generation of reactive oxygen species (ROS) and have been implicated in the pathogenesis of various diseases including cardiovascular disease, diabetes, cancer, and obesity (1). Thus, restoring mitochondrial dysfunction could offer a promising therapeutic approach for such prevalent diseases. This “mitochondrial medicine” requires a fundamental understanding of mitochondrial genetics, oxidative phosphorylation (OXPHOS), ion channels, mechanisms of ROS generation, and the role of mitochondria in the pathogenesis of disease.

Unlike nuclear DNA (nDNA), mitochondrial DNA (mtDNA) is present in multiple copies and is maternally inherited (2). The human mitochondrial genome is only 16.6 kb in size and contains 37 genes. 13 of the genes encode proteins of the OXPHOS complex and the remaining 24 (2 ribosomal, 22 tRNA-encoding) are used to translate proteins (2, 3). Since the discovery of mtDNA in 1963 (4), the importance of the mitochondrial genome has been greatly reinforced by numerous reports highlighting its involvement in several neuromuscular diseases (5–7). For example, mitochondrial gene deletions have been linked to myopathies and neuropathies (8) and certain mitochondrial DNA variants have been implicated in aging and senescence (8, 9).

The identification of pathogenic mtDNA variants has greatly expanded with the development of cutting-edge cell biology and next-generation sequencing techniques. Whole exome sequencing has shown that certain mitochondrial disorders are due to alterations in proteins involved in OXPHOS processes, or others needed for the assembly of these protein complexes (2, 10). While it is well documented that mitochondrial disease occurs in at least 1 in 5000 individuals, the prevalence of pathogenic mtDNA variants may be 1 in 200 as observed by umbilical cord blood screening from newborns, including the ten most common variants (11, 12). Phenotypically, mitochondrial diseases present with multisystem disorders, such as sensory organ failure, myopathies, cardiomyopathies, and neurodegeneration in the adult (13). However, their inheritance can be complex given mitochondrial variation.

Mitochondrial DNA varies in two distinct ways, which is commonly referred to as mitochondrial heterogeneity (14). There can be variation of mtDNA sequence within a single cell, termed heteroplasmy, and variation of mitochondria in different cells of the same organism. Importantly, mitochondrial heterogeneity is regulated via genetic and non-genetic (e.g., metabolic) mechanisms (14). Genetic sources of mitochondrial heterogeneity include changes in mtDNA copy number, mtDNA variants, and loss of mtDNA content mostly due to ROS (14, 15), which can impact the levels of mitochondrial RNA transcripts necessary for the respiratory output of the mitochondrion. The non-genetic mechanisms entail altered structure of electron transport chain (ETC) proteins and the mitochondrial network, disrupted composition of the mitochondrial membrane, and compromised membrane potential. These two mechanisms are inextricably linked. For example, altered transcription, a genetic mechanism of heterogeneity can impact respiratory output, a non-genetic mechanism of heterogeneity.

Interestingly, non-genetic mechanisms (e.g., metabolic state) can also impact the genetic state of a mitochondrion (including mtDNA content) when certain variants that alter mitochondrial structural or functional integrity are selectively targeted. This is possible due to the dynamic nature of the mitochondrial network, with fusion and fission events contributing to the turnover of mtDNA. Mitochondria may exhibit selective fusion and non-selective mitophagy (i.e., mitochondria that are not fused are more likely to undergo mitophagy). Reduced mitochondrial proton gradients may decrease fusion of these mitochondria and thus result in mitophagy. Therefore, if certain mtDNA variants are more likely to alter this gradient, they will be excluded by negative selection via reduced fusion and non-selective mitophagy, thereby promoting retention of variants that promote appropriate membrane potential integrity. Thus, the dynamic nature of the mitochondrial network can impact the mitochondrial genetic state (14, 16).

## Mitochondrial Diseases Caused by Genetic Disruption of OXPHOS Processes

**Figure 1:**
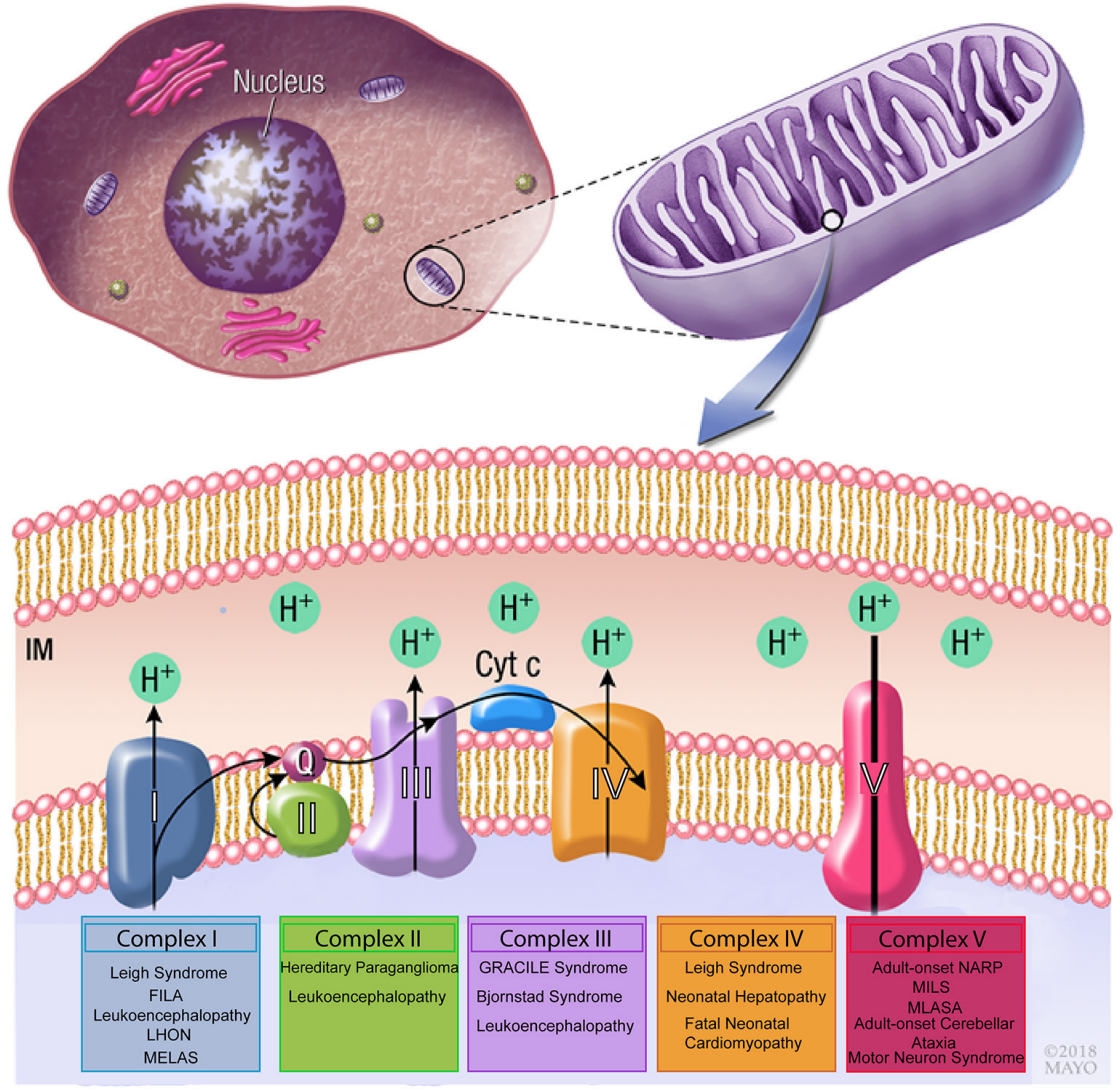
Schematic drawing of the mitochondrial respiratory chain. Complex I, II, III, and IV are essential to generate a proton gradient that is utilized by the ${\text{F}}_{0}^{-}$F_1_-ATP synthase complex to generate ATP. Variants in mitochondrial genes involved in each of the complexes have been associated with neuromuscular disorders. FILA, Fatal infantile lactic acidosis; LHON, Leber hereditary optic neuropathy; MELAS, Mitochondrial encephalomyopathy, lactic acidosis, and stroke-like episodes; GRACILE, growth retardation, aminoaciduria, cholestasis, iron overload, lactic acidosis and early death; MILS, maternally inherited Leigh syndrome; MLASA, mitochondrial myopathy, lactic acidosis, and sideroblastic anemia.

Pathogenic mtDNA variants have been implicated in disease, as alterations in both mitochondrial and nuclear genes affect OXPHOS process of the mitochondrial respiratory chain (17). This is especially clear with variants that impact complex I (also termed NADH:ubiquinone oxidoreductase), which is the largest respiratory chain enzyme and a major contributor to mitochondrial disorders when disrupted (17). Complex I defects occur mainly due to variants in the 44 genes (both in nuclear or mitochondrial genomes) encoding subunits of the complex or proteins involved in its assembly (Figure 1). Examples of these can be seen in Leber hereditary optic neuropathy (LHON), Leigh syndrome and various other mitochondrial diseases. LHON is the most common mtDNA disorder and occurs as a result of homoplasmic variants in one of three genes encoding complex I subunits, m.11778G>A in NADH dehydrogenase 4 (ND4), m.3460G>A in ND1 and m.1448T>C in ND6. Leigh syndrome has also been associated with variants in genes encoding subunits of complex I (18, 19), (Figure 1). Secondary causes causing complex I dysfunction include variants in genes that encode proteins related to the complex's function, such as iron-sulfur cluster assembly and coenzyme Q10 synthesis (17).

Moreover, a great deal of phenotypic diversity is observed between different variants in mtDNA. For instance, the MT-ND4 variants m.11778G>A (p.Arg340His) and m.11777c>A (p.Arg340Ser) have different substitutions for the same amino acid, but are associated with LHON and Leigh syndrome, respectively (17). While in some cases the reason for this discrepancy is unclear (17), in others it may be linked with tissue-specific heteroplasmy as seen with the m.11777c>A (p.Arg340Ser) variant where heteroplasmy levels vary, with reduced variant levels in the brain compared to the skeletal muscle (20). Furthermore, similar phenotypic variation has been observed with NDUFS6 variants, whereby different variants result in a variety of phenotypes including lactic acidosis (c.344G>A), Leigh syndrome (c.3095G>A), and mitochondrial complex I deficiency (c.186+2T>A) (17, 21–23).

While over 250 distinct disease-causing variants have been identified in mtDNA (24), the most common point mutation results in an A to G transition at nucleotide 3243 in the tRNA Leu (UUR) gene with the recurrence of 16 in 100,000 people in northern Finland to a prevalence of 6.2 in 100,000 in Australia (18, 25). This variant typically exhibits high levels of heteroplasmy and causes mitochondrial encephalopathy, lactic acidosis, cardiomyopathy, and stroke-like episodes (MELAS), a multisystem disorder that primarily involves the brain, muscles and endocrine system (12, 26–28). It has also been associated with maternally inherited diabetes and deafness (MIDD) (28, 29). Other examples of common point mutations in mtDNA include A to G transition at nucleotide 8344 in tRNA Lys, which causes myoclonus epilepsy and ragged red fibers (MERRF) syndrome, along with the aforementioned T to G transversion at position 8993 in ATP6, resulting in neuropathy, ataxia, retinitis pigmentosa (NARP) and maternally inherited Leigh syndrome (MILS). All these three pathogenic variants are considered hallmarks of mitochondrial disorders that cover the range of morphological, biochemical, and clinical presentations associated with mitochondrial biology dysfunction (18).

**Figure 2:**
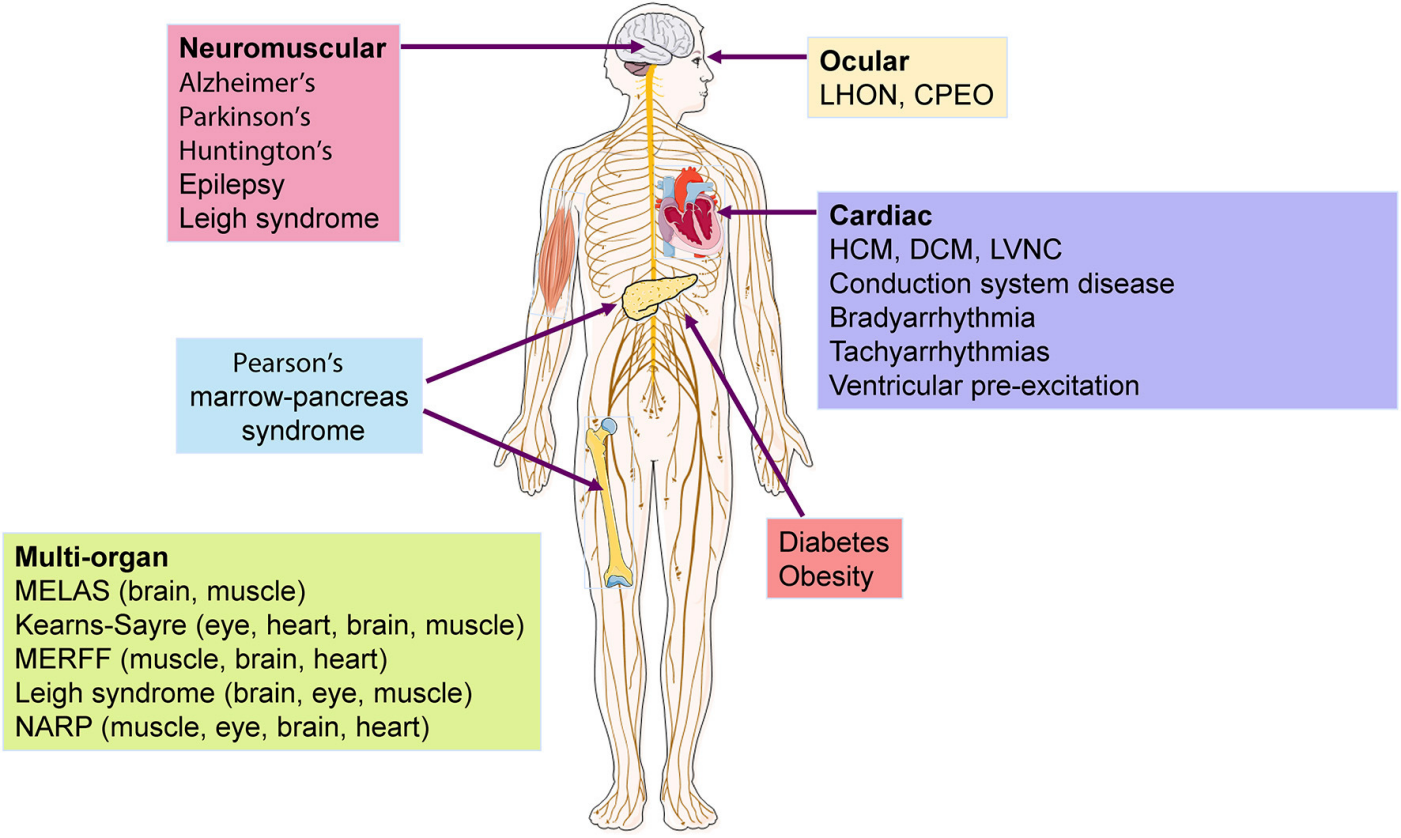
Diseases and organs affected by mtDNA mutations. Several neuromuscular and cardiac disorders have been associated to mtDNA mutations, with some syndromes showing multisystemic incidence that affect mostly the brain, eye, and muscle. LHON, Leber hereditary optic neuropathy; CPEO, chronic progressive external ophthalmoplegia; HCM, hypertrophic cardiomyopathy; DCM, dilated cardiomyopathy; LVNC, left ventricular non-compaction; MELAS, mitochondrial encephalopathy, lactic acidosis, cardiomyopathy and stroke-like episodes ; MERFF, myoclonus epilepsy and ragged red fibers syndrome ; NARP, neuropathy, ataxia, retinitis pigmentosa.

In addition to neuromuscular disorders, mitochondrial dysfunction is associated with more common and complex pathologic conditions, including cardiac disease (30, 31), cancer (32), diabetes (33), Parkinson's disease (34), Alzheimer's disease (35), epilepsy (36), Huntington disease (37), and obesity (38, 39) (Figure 2). In particular, mtDNA variants and reduction in content have been widely involved in cardiac disorders (31, 40), as cardiomyocytes (CMs) have higher mtDNA copy number per diploid nuclear genome (41), given their dependence on OXPHOS to meet high energetic demands. It is therefore unsurprising that a deficiency of OXPHOS leads to mitochondrial dysfunction which can trigger cardiovascular disease (42).

Functional mitochondria are important for cardiomyocyte energy regulation, Ca^2+^ homeostasis, and physiological inflammatory homeostasis. The role of cytosolic [Ca^2+^] to activate cardiac muscle contraction and ATP production via mitochondria is well-established (43), as mitochondria maintain intracellular calcium homeostasis and match energetic demand through the mitochondrial calcium uniporter (MCU) channel. Importantly, *Mcu* knockout mice display no overt baseline phenotype and are protected against mitochondria Ca^2+^ overload in an *in vivo* myocardial ischemia-reperfusion injury model, by preventing the activation of the mitochondrial permeability transition pore, decreasing infarct size, and preserving cardiac function (44). Additionally, *Mcu*^/^ mice exhibit reduced contractile responsiveness to acute β-adrenergic receptor stimulation and in parallel are unable to activate mitochondrial dehydrogenases, displaying delayed matching of energy output to adrenergic or functional demand. These results support the hypothesis that MCU may be dispensable for homeostatic cardiac function but required to modulate Ca^2+^- dependent metabolism during acute stress (44). Moreover, the deletion of *Mcu* greatly decreases susceptibility to mitochondrial permeability transition pore (MPTP) activation and thereby provides protection against necrotic cell death. Additional studies have revealed the relation between mitochondrial calcium content and cardiac dysfunction, suggesting a potential role for mitochondrial dysfunction in the pathophysiology of cardiac disorders, as reviewed in (45).

## Disease Modeling and Drug Screening of Mitochondrial Disorders Impacting the Cardiovascular System

Common barriers and limitations in current drug discovery and development include the cost and low sensitivity of non-human animal models for the study of off-target toxicities (e.g., QT prolongation), and limited availability of human CMs (46). Due to genetic and physiological similarities to humans regarding the effect of mitochondrial dysfunction on post-mitotic tissues, mouse models have been extensively used to model mitochondrial disorders, being advantageous over cell lines and/or organoid cultures (47). In 1995 there were almost 50 mice strains expressing transgenes encoding mitochondrial proteins, with almost half of these being associated with mitochondrial diseases (47, 48). However, using mice as a model for mitochondrial dysfunction has considerable disadvantages as they require a high level of maintenance and do not always recapitulate human phenotypes, as shown by striking differences in muscle fiber excursion during walking, critical to model neuromuscular diseases such as Duchenne muscular dystrophy (49). Moreover, species-differences relative to human cardiac physiology include beating rate (typically slower in humans), energetics, myofilament configuration, myosin heavy chain isoform expression, presence of ion channels and electrophysiology, and Ca^2+^ cycling (50). Thus, mice models may not be adequate for the early screening of a large number candidate compounds (47) to treat mitochondrial disorders.

As mtDNA variants impact preferentially in the heart, an abundant and physiologically-relevant platform to model cardiovascular diseases is needed. The clinical investigation and application of primary human CMs are further limited by donor cell availability and problematic isolation procedures (51). Given the lack of immortalized cardiomyocyte cell lines and the difficulty of obtaining appropriate animal models of advanced cardiac disease, development of new heart disease-specific therapeutics would benefit tremendously from advances in human induced pluripotent stem cell (hiPSC)-derived cardiomyocyte (CM) technologies. Importantly, these cells have overcome some of the limitations of animal models by providing a virtually infinite and physiologically-relevant source of CMs that have been extensively characterized *in vitro* in terms of molecular and functional features (52).

Molecular profiling of hiPSC differentiation into a CM lineage involves the serial activation of distinct genes that constitute the hallmark of normal cardiac development. *In vitro* differentiation initially is characterized by expression of (i) BRY and MIXL1 to form the mesoderm, (ii) MESP1, ISL1, and KDR to design cardiogenic mesoderm and (iii) NKX2.5, GATA4, TBX5, MEF2C, and HAND1/2 expressed in cardiac-specific progenitors stage (50). Finally, structural genes encoding for sarcomeric-related proteins such as MYL2, MYL7, MYH6, and TNNT2 are expressed in terminally differentiated CMs (50, 53–55). Altogether, the key goal for recapitulating cardiovascular development to boost differentiation efficiency is based on modulating signaling pathways such as Wnt, BMP, and Activin/Nodal/TGF-β (56–59). Therefore, the hiPSC-derived cardiac progenitor cells (CPCs), and CMs offer possible ways to address new drugs to market.

Moreover, a key feature of hiPSCs involves their patient-specific nature, thus providing a model system supporting personalized medicine approaches. The “patient-in-a-dish” from iPSCs approach has exhibited great potential to contribute to a better understanding of the exact pathological mechanisms of rare diseases (60). Further advances in hiPSC-CM technology have facilitated the study of pathophysiology and drug efficacy in 3D organoid environments with an expandable supply of cells from donor patients (61). This confers the benefits of using hiPSC-CMs but provides additional physiologically-relevant conditions experienced at the organ level. After screening candidate compounds using hiPSC-CM cells, *in vivo* testing can be pursued (61) on a more reduced number of drugs, thus limiting the risk and cost. This approach can address the limited applicability of mouse models for drug discovery by providing a translational system that enables screening of a large number of candidate compounds to treat mitochondrial diseases.

Overall, all of these desirable properties make pluripotent stem cell-based models a promising platform for drug testing and toxicology screening (46, 62–65). Additionally, hiPSC-CMs serve as a valuable model for pre-clinical screening of candidate anti-arrhythmic and anti-heart failure pharmacological agents, as well as studying the off–target cardiac toxicities of chemotherapeutic agents (66–68). However, there is still room for improvement of this cellular model as iPSC-derived lineages are typically immature relative to adult counterparts, and they fail to recapitulate multi-cellular organs with neurohormonal control (69). Nevertheless, there has been considerable progress in refining hiPSC-CM maturation (70), which have contributed significantly to cardiovascular research and has been applied to model several disorders. For example, hiPSC-CM have successfully modeled familial dilated cardiomyopathy (DCM) (71), catecholaminergic polymorphic ventricular tachycardia (CPVT) (72), and familial hypertrophic cardiomyopathy (HCM) (73). Thus, iPSC-CM technology greatly facilitates the study of genetic cardiovascular diseases, development of cardiovascular system, toxicological screening, drug discovery, and personalized cell-based therapy (50).

While these cardiomyopathy hiPSC-CM models focused mostly on mutations in sarcomeric genes that regulate cardiomyocyte contraction and calcium handling, a few have also showed energy depletion phenotypes due to mitochondrial dysfunction (74, 75). Importantly, hiPSC-CMs have also been harnessed to specifically model mitochondrial cardiomyopathies as these constitute phenocopies of HCM (40). Wang et al. derived a number of hiPSC lines from Barth syndrome patients showing frameshift or missense mutations in the Tafazzin (TAZ) gene (76). Human iPSC-CMs differentiated from these lines have demonstrated several disease phenotypes in comparison to healthy isogenic controls, such as reduced mitochondrial respiration activity, impaired sarcomere organization and decreased contractile stress generation in a tissue construct. These phenotypes were mechanistically linked with increased formation of ROS and immature cardiolipin.

Currently, most studies relating mutations in mtDNA to cardiovascular disorders rely on large-scale mitochondrial genetics to associate specific variants with patient cohorts exhibiting different cardiac phenotypes (31). While this approach is statistically robust, it lacks functional characterization of pathological phenotypes exhibited by cardiomyocytes *in vitro*, required to better understand disease progression and treatment. To the best of our knowledge, only one study characterized the impact of a mtDNA mutation that associated with HCM (77) in hiPSC-CMs. Li and colleagues have generated hiPSCs from HCM patients bearing the m.2336T>C mutation in the mitochondrial rRNA gene (MT-RNR2). When compared to unrelated healthy controls, diseased hiPSC-CM exhibited markedly lower levels of several mitochondrial proteins (MT-ND5, MT-CYB, MT-COX2, MT-ATP8), resulting in unstable 16S rRNA and ultrastructure defects in the mitochondria. Strikingly, these alterations led to several phenotypes characteristic of HCM, such as reduced ATP/ADP ratio and mitochondrial membrane potential as well as abnormal calcium handling (e.g., increased intracellular Ca^2+^ levels). This study not only strongly supported causation of HCM by mtDNA mutations, but also overcame limitations associated with clinical studies showing varying tissue-specific heteroplasmy and susceptibilities to specific mtDNA mutations (78). Furthermore, our own studies related different *in vitro* phenotypic severities between hiPSC-CM lines bearing either the R453C-βMHC or the E99K-ACTC1 sarcomeric mutations with specific variants in mtDNA (79). This approach focused on coupling phenotypes of hiPSC-CMs with mtDNA sequencing is promising to unveil novel variants with potentially HCM-protective or aggravator function.

Remarkably, it is possible to generate iPSC-CMs clones representing a range of both healthy and diseased mtDNA for the study of mitochondrial disease. Moreover, producing independent subclones that have distinctive heteroplasmic mtDNA patterns in the context of native nuclear DNA enables deconvolution of authentic disease specific processes. For example, it has been previously demonstrated that MELAS-iPSC clones show a similar range of mtDNA heteroplasmy of the disease-causing variants as the original patient derived fibroblasts. Producing MELAS-iPSC clones with high and low levels of heteroplasmy and differentiating them along a cardiac lineage enabled direct comparison of genotype/phenotype relationships to investigate the impact of mutant mtDNA on MELAS patients (80). Additionally, our group has shown that iPSCs are capable of modeling intra- and inter-person variability stemming from different levels of heteroplasmy in mutant mtDNA between iPSC clones, including the functional consequences for mitochondrial respiration in iPSC-CMs (81). Overall, this results in a platform that be used to investigate pharmacological approaches for reducing the burden of mutant mtDNA. In addition, hiPSC-CM may also overcome the limitations of clinical mitochondrial genetic studies, where patient-derived samples are mostly collected from peripheral blood and therefore do not reflect the tissue-specific heteroplasmy showed by CMs [requiring clinically invasive procedures to harvest (78)].

Notwithstanding, hiPSC-CM technology is very recent and still needs to be further developed to become an ideal platform for the study of pharmacology, toxicology, pathogenesis, and cell-based therapy (60), although a number of methodological improvements have been published (82, 83). In particular, the investigation of mitochondrial diseases and recapitulation of mitochondrial dysfunction phenotypes will greatly benefit from metabolic maturation strategies. In fact, while the adult heart preferentially relies on fatty acid oxidation to sustain high energetic demands (84), hiPSC-CMs more closely resemble fetal heart metabolism by primarily depending on glycolysis (85). In order to bridge this gap, several hiPSC-CM maturation media were developed, consisting of supplementation with several fatty acids including palmitate, oleate and linoleic acid (86–88). Additionally, the inclusion in the media of fatty acid transporters into the mitochondria such as L-carnitine, or inhibition of lactate dehydrogenase A has further enhanced the switch from glycolysis to OXPHOS. Importantly, these different media formulations have consistently resulted in increased mitochondrial respiration capacities, mitochondrial content and cellular ATP levels, as well as functional improvement of cardiomyocyte calcium handling, ultrastructural features and contractility. Alternatively, transition from 2D monolayers to 3D aggregate cultures under agitation demonstrated changes in the transcriptome of hiPSC-CMs, leading to upregulation of genes involved in OXPHOS at the expense of glycolytic genes. These changes were reflected by lower glycolytic fluxes in 3D, accompanied by an increased TCA cycle activity, as measured by 13C-based metabolic flux analysis (89). Altogether, metabolic maturation strategies have effectively surpassed initial limitations associated with fetal-like metabolism of hiPSC-CMs.

Overall, this new technology will greatly complement current cell and animal models, and holds great promise in providing insight into the drug discovery, with new tools including biosensor photoproteins (90) and a strong predictive advantage for moving compounds into clinical practice (91).

## Mitochondrial Maintenance and Nicotinamide Adenine Dinucleotide (NAD^+^) Levels

NADH, along with its oxidized form NAD^+^, are fundamental cofactors in energy metabolism. NAD^+^ in eukaryotic and prokaryotic cells is primarily synthesized from tryptophan or through the salvage pathway, which uses nicotinic acid and nicotinamide as precursors (92, 93). Since mitochondrial membranes have shown impermeability to NAD^+^ and NADP, two major pools of NAD^+^ and NADP in cells have been found, in the cytoplasm and mitochondria (94, 95). The cytosolic NAD^+^/NADH redox cycling and homeostasis are maintained by transporting the cytosolic NADH into the mitochondria through the malate aspartate shuttle or the glycerol phosphate shuttle (96). Previous studies have reported that the total intracellular NAD^+^: NADH ratio is about 3–10^4^. However, the ratio of the free NAD^+^/NADH form is a more reliable indicator of cellular redox potential compared to the ratio of total NAD^+^/NADH (97). Under the physiological conditions in typical eukaryotes, the cytosolic free NAD^+^/NADH ratio is about 60–700 (98–100), while the ratio of mitochondrial NAD^+^/NADH is between 4 and 10 (97, 98, 101). For example, in a mouse model of transverse aortic constriction (a model of pressure overload) the NAD^+^/NADH ratio is around 2.75 (102). Investigation of cellular metabolism associated with NAD^+^/NADH redox state is essential in both healthy and disease circumstances.

It has been well-established that increased levels of NAD^+^ and sirtuin activation play a critical role in regulating mitochondrial homeostasis and lifespan (103). Sirtuins are a family of deacetylases that use NAD^+^ as a cofactor and mediate mitochondrial homeostasis. For example, activation of SIRT1 and subsequent deacetylation and activation of peroxisome proliferator-activated receptor gamma coactivator-1alpha (PGC-1α), a coactivator of mitochondrial biogenesis, promotes increased ATP production. SIRT1 also activates forkhead box protein O1 (FOXO1) which increases fatty acid oxidation. Additionally, activation of SIRT3, a mitochondrial sirtuin, promotes fatty acid oxidation and is protective against reactive oxygen species. Given the role of NADH in oxidoreductive reactions of glycolysis, the Krebs cycle, fatty acid oxidation, and oxidative phosphorylation, alterations in NADH/NAD^+^ can have broad metabolic effects (104, 105). In fact, a decreased NAD^+^/NADH ratio is strongly associated with mitochondrial and age-related disorders including cancer, obesity, neurodegeneration, and diabetes (106–109). The level of NAD^+^ decreases with age in multiple models including worms and rodents as well as human tissue (107, 110–112). Research demonstrated that increasing the level of NAD^+^ leads to NAD^+^/sirtuin pathway activation and subsequent effects on multiple metabolic pathways. For example, treating the cytochrome *c* oxidase (COX) deficiency indicative of mitochondrial disorder with the AMPK agonist 5-aminoimidazole-4-carboxamide ribonucleotide (AICAR) partially rescued mitochondrial dysfunction and improved motor outcomes (113). Thus, regulation of mitochondrial metabolism via evolutionarily conserved NAD^+^/sirtuin pathways presents a novel target for clinical trials.

Recent evidence suggests that NAD^+^ and PARP inhibitors could be used to boost NAD^+^ levels in cell culture and animal models (107). Moreover, additional work has shown that, in *Caenorhabditis elegans* and mice, α-amino-β-carboxymuconate-ε-semialdehyde decarboxylase (ACMSD) controls cellular NAD^+^ levels. ACMSD is an enzyme that plays a role in *de novo* NAD^+^ synthesis pathways by limiting spontaneous cyclization of α-amino-β-carboxymuconate-ε-semialdehyde. Interestingly, not only genetic inhibition of ACMSD but also the pharmacological inhibition of ACMSD increases *de novo* NAD^+^ synthesis and sirtuin 1 activity (103), resulting in enhancement of mitochondrial function (103). Moreover, in addition to aging, an altered NAD^+^/NADH ratio is observed in cardiac disease. Specifically, a decreased utilization of NADH may result in a reduced NAD^+^/NADH ratio observed in failing hearts suggesting an inability to maintain NADH production due to mitochondrial dysfunction (12, 13, 114, 115). Both pharmacological and genetic attempts to increase NAD^+^ levels and subsequently the NAD^+^/NADH ratio have resulted in improved cardiac function in mouse models of heart failure (13, 115–117).

In 1924, Otto Warburg proposed that the energy in cancer cells is produced by a shift from oxidative phosphorylation to aerobic glycolysis (118), dramatically increasing the biosynthesis of macromolecules for rapid cell proliferation (32, 119, 120). Classical biochemical techniques including chromatography, mass spectrometry, enzymatic cycling assays, and nuclear magnetic response spectroscopy are not applicable methods for performing quantitative, high-throughput screening in real-time. As the NAD^+^/NADH ratio plays a central role in all aspects of cellular metabolism, real time tracking of this metabolic state in living cells needs to be developed. Previous techniques relied upon the weak endogenous fluorescence of NADH, examined by single-proton or multiphoton excitation for measuring metabolic states of mitochondria (96, 97, 121, 122). However, these methods are plagued by innate disadvantages, including limited sensitivity and resultant cellular injury associated with ultraviolet irradiation (123). Because most of the NADPH fluorescence derives from the mitochondria, it is often challenging to identify and separate the bright mitochondrial signals from those emanating from the cytosol. Moreover, it is difficult to distinguish NADPH from NADH, as they are spectrally identical. Recently developed technology employing fluorescence lifetime imaging can quantitatively differentiate between the two cofactors based on the fact that bound NADH and bound NADPH acquires different fluorescence lifetimes inside the cell (123). Nevertheless, usage of fluorescence lifetime imaging is neither technically simple nor broadly applicable as it requires the separation of NADH and NADPH redox signaling without disrupting the samples on the addition of external probes.

Enzymatic cycling assays, chromatography and mass spectrometry are a few of the conventional methods that are often used to measure the intracellular NAD^+^/NADH redox state (97). Additional limitations of these techniques involve the time required to conduct the assays, and their incompatibility with the study of spatiotemporal dynamics in single, intact cells, thereby making them unsuitable for quantitative, real-time high-throughput screening in living cells (97, 124, 125). To overcome the challenges of NAD^+^/NADH dynamics analysis with subcellular resolution *in vivo*, we propose a new technology using a genetically encoded fluorescent sensor based on fluorescent proteins (FPs) with the ability to analyze NAD^+^/NADH dynamics with subcellular resolution.

## Live Monitoring of NAD^+^/NADH Redox State in Real Time by Genetically Encoded Sensor Technology

Due to the limitations of conventional methods explored above, genetically encoded fluorescent sensors may present an adequate alternative for live monitoring of NAD^+^/NADH redox state, supporting rapid and efficient metabolic chemical screening. Developing these sensors involves single-cell, real-time monitoring of multiple metabolic parameters (120). Recently, two independent groups have developed genetically encoded NADH sensors: Peredox and Frex (126, 127). In 2016, the Yang group described a second-generation genetically encoded biosensor for NAD^+^/NADH, named Sensor of NAD (H) redox (SoNar) (128). All three sensors quantify NADH cellular levels or the NAD^+^/NADH ratio through specific, non-covalent binding resulting in a conformational change that alters the fluorescent properties of the sensor. Since these proteins are genetically encoded and have intrinsic fluorescence without extraneous compounds, they can be easily introduced into live cells via DNA transfection and targeted to specific organelles. However, these proteins vary in their fluorescent properties. Notably, Frex and SoNar have two excitation peaks (97), enabling determination of ratiometric fluorescence. Thus, these can be used for detecting the NAD^+^/NADH ratio given the differential effect of NAD^+^ or NADH on the two excitation wavelengths. On the other hand, Frex can be used to detect NADH levels independent of sensor expression levels (97, 129). In contrast, Peredox must be made ratiometric by using fusing it with mCherry (97).

Compared to the first-generation sensors, SoNar provides a significant improvement for live cell NAD^+^/NADH measurement (97) (Table 1). Given its shorter coding sequence, SoNar has a more intense fluorescence enabling its use for *in vivo* applications compared to Frex (128). SoNar has a wide dynamic range and high intensity fluorescence. It is rapidly responsive and thus suitable for tracking subtle changes of cellular metabolic and redox states *in vivo*. This sensor represents an improved reporter system for studying cell metabolism (130, 131) and compounds for drug discovery. In contrast to other available assays, which target a single protein or enzyme, SoNar is even capable of reporting several pathways affecting energy metabolism providing a more detailed insight into glycolysis and mitochondrial respiration (128). Previously, it was difficult to rigorously measure NAD^+^/NADH levels in certain cancer lines. However, using SoNar, MDA-MB-468, U87, and H1299 cells, were shown to have a significantly reduced NAD^+^/NADH cytosolic ratio (132).

**Table 1:** SoNar has some key advantages for live cell monitoring of NAD^+^/NADH compared to Frex and Peredox-mCherry.

	**Highlights**	**Limitations**	**Detected Signal**
**Peredox-mCherry**	Modest brightness in cells Aggregates in cytosol	Low dynamic range Large gene Not validated *in vivo*	NAD^+^/NADH ratio^*^
**Frex**	Does not aggregate in cells Moderate dynamic range	Minimal brightness in cells Large gene	NADH
**SoNar**	High brightness in cells High dynamic range Small gene Does not aggregate in cells Validated *in vivo*	pH sensitive at 485 nm	NAD^+^/NADH ratio

* *However, it is impacted by absolute NAD(H) levels*.

However, the use of the SoNar sensor still poses a number of challenges. SoNar fluorescence may be impacted by changes in pH. While the chromophore responsible for fluorescence absorption at 420 nm is protonated and not sensitive to pH changes, the chromophore operating at 485 nm is normally deprotonated and responsive to changes in pH, which can trigger protonation and block fluorescence emission. This can be compensated by using absorption only at 420 nm, although this will impact the dynamic range of SoNar (97). Additionally, this sensor does not allow for the absolute quantification of NADH or NAD^+^ but provides a measure of the NAD^+^/NADH ratio, which is the key value that changes in disease state. Furthermore, the genetic construct encoding the sensor needs to be integrated into the host cell's genome, preventing its use for staining active live clinical samples (97). Nonetheless, this strategy can be harnessed to generate stable hiPSC reporter lines tracking NAD^+^/NADH ratios that can then be differentiated into CMs to investigate metabolic cardiovascular disorders such as mitochondrial cardiomyopathies (133). This can be achieved by knocking-in the genetic construct into safe loci such as *AAVS1*, as previously demonstrated with genetically encoded calcium sensors (75). The properties of SoNar sensors, displaying high fluorescence intensity and dynamic range, are compatible with live imaging, enabling their application for high-content imaging, and/or for the measurement of overall signal intensity in a standard plate reader. Additionally, treatment with modulators of redox state in hiPSC-CMs such as hydrogen peroxide and DTT can be done to establish extremes of oxidation and reduction states of cellular NAD^+^/NADH, respectively (134), leading to a more accurate quantification of the signal in the context of mitochondrial diseases where this ratio is disrupted (132). Moreover, these approaches can be multiplexed with existing platforms for evaluating mitochondrial respiration profiles in hiPSC-CMs such as the Seahorse assay (82), which is performed in 96-well plate format thereby facilitating high-throughput screening studies, in response to various energy sources or metabolic modulators such as perhexiline (51). This approach may also be incorporated in isogenic sets of patient-derived hiPSC lines with known mitochondrial diseases affected by mtDNA variants (79).

## Conclusions

**Figure 3:**
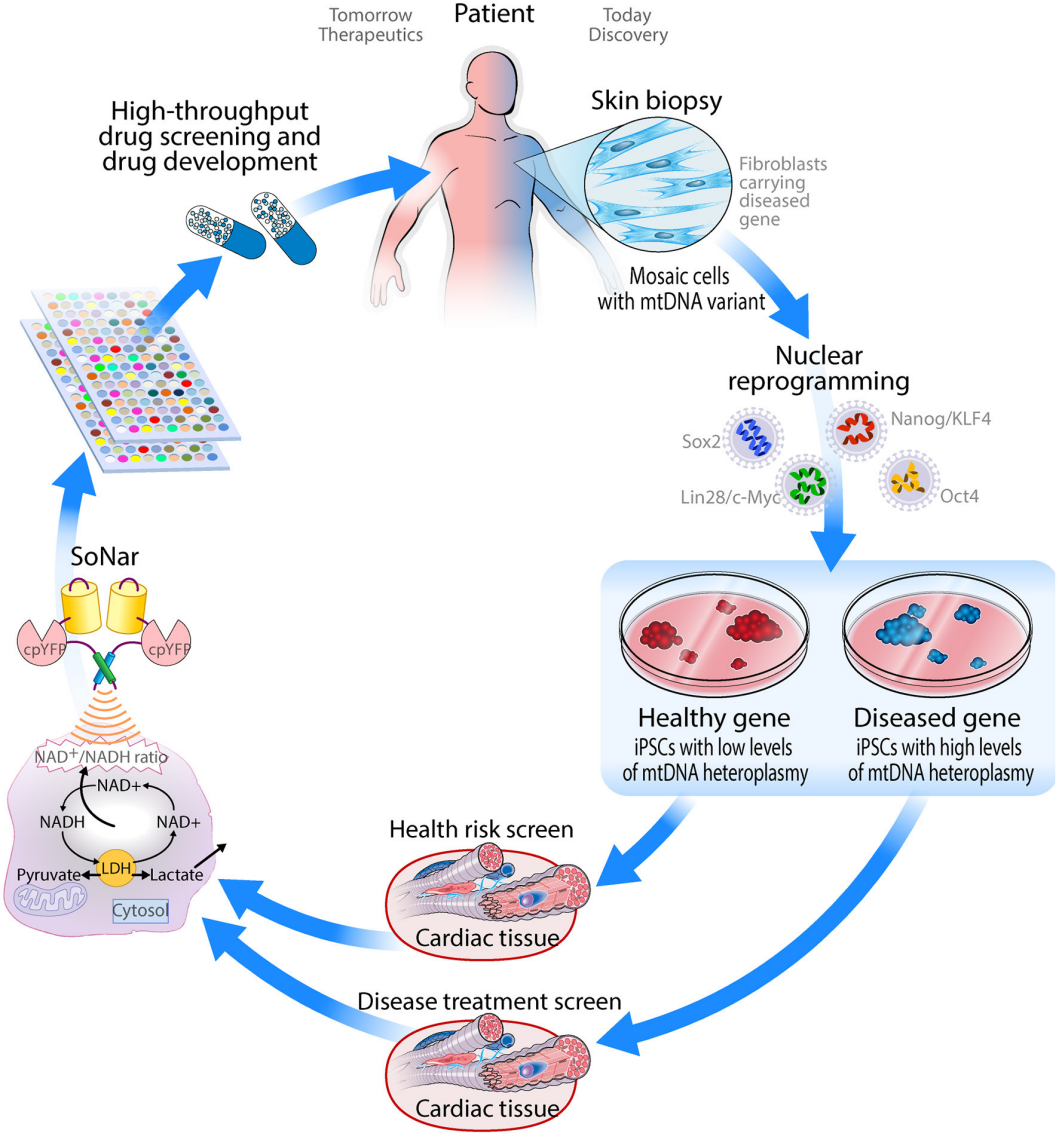
Schematic drawing of how iPSC-derived cardiomyocytes can be applied as a drug screening platform for mitochondrial disorders.

Disruption of mitochondrial function is not only commonly observed in cardiovascular disorders but has also been proposed to underlie the pathology of disease progression (135), due to the high energetic demand of the heart and its cellular constituents. In turn, mitochondrial DNA variants have shown to cause phenocopies of cardiomyopathies (31), highlighting the close relationship between mitochondrial dysfunction and cardiovascular disease. Herein, we propose that human iPSC-derived cardiomyocytes provide a unique translational model system to advance understanding of mitochondrial pathogenic variants. These cellular models have the potential for not only investigating mitochondrial dysfunction caused by mtDNA variants (such as m.3243A>G involved in MELAS), but also as a drug screening platform for both mitochondrial and cardiovascular disorders. The genetically encoded fluorescent SoNar sensor that tracks NAD^+^/NADH ratio provides a robust tool for quantifying the intracellular redox state and screening for small compounds that restore normal metabolic activity. Altogether, the combination of iPSC-CMs with the SoNar sensor is expected to transform the future treatment of metabolic and cardiovascular diseases by supporting the discovery of drugs treating these inextricably linked conditions (Figure 3). Finally, these cellular models can provide a platform for optimization of recently discovered tools to edit the mitochondrial genome (136), by offering a disease-relevant pathophysiology setting in the cardiac cell type of interest, more accurately recapitulating heart-specific heteroplasmy.

## Author Contributions

PK conducted the literature search and compiled references into a cohesive draft. DM and TK carried out review, editing, and final approval of the manuscript.

## Conflict of Interest

The authors declare that the research was conducted in the absence of any commercial or financial relationships that could be construed as a potential conflict of interest.

## Abbreviations

iPSCs: induced pluripotent stem cells
CMs: Cardiomyocytes
iPSC-CM: induced pluripotent stem cell-derived cardiomyocyte
ESCs: embryonic stem cells
mtDNA: mitochondrial DNA
nDNA: nuclear DNA
MELAS: Mitochondrial encephalomyopathy, lactic acidosis, and stroke-like episodes
OXPHOS: Oxidative phosphorylation
ETC: Electron transport chain
ATP: Adenosine triphosphate
NADH: Nicotinamide adenine dinucleotide
SoNar: Sensor of NAD (H) redox.
